# The relationship of preoperative estimated glomerular filtration rate and outcomes after cardiovascular surgery in patients with normal serum creatinine: a retrospective cohort study

**DOI:** 10.1186/s12871-019-0763-1

**Published:** 2019-05-29

**Authors:** Myung-Soo Jang, Jae-Sik Nam, Jun-Young Jo, Chang-Hwa Kang, Seung Ah. Ryu, Eun-Ho Lee, In-Cheol Choi

**Affiliations:** 1 Department of Anesthesiology and Pain Medicine, College of Medicine, Kyung Hee University, Seoul, South Korea; 2 Department of Anesthesiology and Pain Medicine, Laboratory for Perioperative Outcomes Analysis and Research, Asan Medical Center, University of Ulsan College of Medicine, 88 Olympic-ro 43-gil, Songpa-gu, Seoul 05505 Korea; 3 Department of Anesthesiology and Pain Medicine, Seoul Medical Center, Seoul, Korea

**Keywords:** Cardiovascular surgery, Glomerular filtration rate, creatinine

## Abstract

**Background:**

Although serum creatinine concentration has been traditionally used as an index of renal function in clinical practice, it is considered relatively inaccurate, especially in patients with mild renal dysfunction. This study investigated the usefulness of preoperative estimated glomerular filtration rate (eGFR) in predicting complications after cardiovascular surgery in patients with normal serum creatinine concentrations.

**Methods:**

This study included 2208 adults undergoing elective cardiovascular surgery. Preoperative eGFR was calculated using Chronic Kidney Disease Epidemiology Collaboration equations. The relationships between preoperative eGFR and 90 day postoperative composite major complications were analyzed, including 90 day all-cause mortality, major adverse cardiac and cerebrovascular events, severe acute kidney injury, respiratory and gastrointestinal complications, wound infection, sepsis, and multi-organ failure.

**Results:**

Of the 2208 included patients, 185 (8.4%) had preoperative eGFR < 60 mL/min/1.73 m^2^ and 328 (14.9%) experienced postoperative major complications. Multivariable logistic regression analyses showed that preoperatively decreased eGFR was independently associated with an increased risk of composite 90 day major postoperative complications (adjusted odds ratio: 1.232; 95% confidence interval [CI]: 1.148–1.322; *P* <  0.001). eGFR was a better discriminator of composite 90 day major postoperative complications than serum creatinine, with estimated c-statistics of 0.724 (95% CI: 0.694–0.754) for eGFR and 0.712 (95% CI: 0.680–0.744) for serum creatinine (*P* = 0.008).

**Conclusions:**

Decreased eGFR was significantly associated with an increased risk of major complications after cardiovascular surgery in patients with preoperatively normal serum creatinine concentrations.

**Electronic supplementary material:**

The online version of this article (10.1186/s12871-019-0763-1) contains supplementary material, which is available to authorized users.

## Background

Several studies have demonstrated that the risk of postoperative complications and mortality is higher in patients with preoperative renal dysfunction than in those with normal renal function [1–6]. Thus, correct identification of preoperative renal function may be important in predicting the prognosis of patients undergoing cardiovascular surgery.

Serum creatinine (sCr) concentration has been regarded as an index of renal function in clinical practice [7], as well as being a component of several perioperative risk prediction models [8, 9]. However, because sCr level can be influenced not only by its renal excretion, but by other factors, including age, gender, ethnicity, muscle mass, and diet, it may remain within a normal range, even in patients with significant renal dysfunction [10, 11]. Estimated glomerular filtration rate (eGFR) has been reported to be a more useful measure than sCr in detecting and evaluating renal dysfunction and in predicting postoperative outcomes [1, 3, 4, 11–14]. Of several equations developed to calculate eGFR so far, the Chronic Kidney Disease Epidemiology Collaboration (CKD-EPI) equation is known to be more accurate and precise than the other equations in estimating GFR, especially in people with high GFR levels [15, 16]. Thus, the 2012 Kidney Disease Improving Global Outcomes (KDIGO) clinical practice guidelines recommend using the CKD-EPI equation to estimate GFR [17]. To our knowledge, however, no study to date has shown that eGFR calculated using the CKD-EPI equation can predict the prognosis of patients with normal sCr undergoing cardiovascular surgery. This study therefore assessed whether eGFR calculated using the CKD-EPI equation can predict major complications and mortality within 90 days after surgery in patients with normal sCr (< 1.4 mg/dl) undergoing cardiovascular surgery.

## Methods

### Study design, participants

All patients aged ≥20 years who underwent cardiovascular surgery in a tertiary hospital in South Korea between July 2012 and July 2015 were included. All baseline demographic and perioperative clinical information were obtained from the Asan Medical Center Cardiovascular Surgery and Anesthesia Database and from a retrospective review of the computerized patient record system (Asan Medical Center Information System Electronic Medical Record), as described [18]. Patients who had undergone preoperative dialysis, those without preoperative sCr measurements, and patients with preoperative sCr concentrations > 1.4 mg/dL (i.e., the upper limit of the normal range at our institution and considered abnormal) were excluded. Also excluded were patients who had undergone emergency or urgent surgery, those with a preoperative intra-aortic balloon pump or ventricular assist device support, and those who underwent endovascular aortic repair surgery. This retrospective study was performed according to the guidelines of the Strengthening the Reporting of Observational Studies in Epidemiology [19] and was approved by the Institutional Review Board of Asan Medical Center (AMC IRB 2017–0593), which waived the requirement for written informed consent because of the observational nature of the study.

### Definitions of variables

The primary outcome was a composite of major complications within 90 days after surgery, including 1) death, 2) a major adverse cardiovascular or cerebrovascular event (MACCE), 3) pulmonary complications, 4) renal complications, 5) wound complications, 6) gastrointestinal complications, 7) sepsis, or 8) multi-organ failure. A patient who experienced more than one of these events was counted only once in the composite outcome. Major complications within 90 days after surgery were defined according to the European Perioperative Clinical Outcome definitions or as previously reported [20–22]. Mortality was defined as death from any cause within 90 days of primary cardiovascular surgery. Major adverse cardiovascular or cerebrovascular events included myocardial infarction, malignant ventricular arrhythmia, cardiac dysfunction, and ischemic or hemorrhagic stroke. Pulmonary complications included pneumonia of any cause, acute respiratory distress syndrome, and respiratory failure requiring mechanical ventilation for more than 48 h. Renal complications included severe acute kidney injury (KDIGO stage ≥ 2, i.e., an increase in sCr to ≥ 2.0 times that of the baseline value within 7 days of surgery) and the need for renal replacement therapy. For the diagnosis of severe acute kidney injury, urine output was not used due to incomplete recording and possible effects of diuretic use. Wound complications included surgical site infection, wound dehiscence, and mediastinitis. Gastrointestinal complications included gastrointestinal hemorrhage, mesenteric ischemia, pancreatitis, and hepatic failure. Secondary outcomes included the incidence of specific individual complications of the primary outcome and composite 30-day postoperative major complications. Data regarding postoperative morbidity and mortality were obtained from visiting outpatient clinics, by a detailed review of all medical records, by telephone interviews, or from the National Population Registry of the Korean National Statistical Office.

Preoperative renal function was assessed using both sCr and eGFR. sCr concentration was routinely checked preoperatively and daily until 3 days after surgery (upon arrival at the ICU, and at 1, 2, and 3 days after surgery); however, after 3 days postoperatively, sCr concentration was ordered at clinician (surgeon or intensive care physician) discretion until hospital discharge. The preoperative sCr concentration was defined as that measured closest to the time of surgery (but within 30 days of surgery). sCr concentration was measured using the kinetic Jaffe method (Cobas® 8000 modular analyzer series; Roche Diagnostics GmbH, Vienna, Austria) which was traceable to standardized reference method (isotope dilution mass spectrometry). Preoperative eGFR was calculated using the CKD-EPI equation (eGFR = 141 × min(sCr/κ, 1)^α^ × max(sCr/κ, 1)^− 1.209^ × 0.993^age^ × 1.018 (if female) × 1.159 (if black), where κ is 0.7 for females and 0.9 for males, α is − 0.329 for females and − 0.411 for males, min indicates the minimum of sCr/κ or 1, and max indicates the maximum of sCr/κ or 1) [15].

### Statistical analysis

As this study was conducted for exploratory purposes, minimum sample size was not calculated and all included patients were analyzed. Categorical variables are presented as numbers and percentages, and continuous variables as mean ± standard deviation or median and interquartile range.

The unadjusted relationship between preoperative eGFR and composite 90-day postoperative major complications was analyzed using descriptive statistics, logistic regression, and receiver operating characteristic (ROC) curve analysis. Preoperative eGFR was analyzed as both a continuous variable and as a categorical variable, arbitrarily classified into groups with eGFR < 60, 60–74, 75–89, 90–104, and ≥ 105 mL/min/1.73 m^2^. Although categorization may be simple and attractive from the perspective of decision making, arbitrary classification may result in loss of information. Therefore, restricted cubic spines were adapted as an alternative for more flexible description of their relationship.

The independent associations between preoperative eGFR and composite 90-day postoperative major complications were evaluated using multivariable logistic regression analyses. In addition to preoperative renal function, all preoperative variables in Table 1 were assessed independently, and variables with a *P* value < 0.20 in the univariate analyses were entered into the multivariable analyses. A backward elimination process with a *P* value cutoff of 0.05 was used to develop the final multivariable models. Additionally, univariate and multivariate analyses were conducted to evaluate the relationships between preoperative eGFR and the secondary outcome variables. Adjusted odds ratio (OR) with 95% confidence interval (CI) for the logistic regression were calculated. Model discrimination and calibration were measured using c-statistics and Hosmer-Lemeshow statistics, respectively.

**Table 1:** Baseline and intraoperative characteristics of study patients stratified by preoperative eGFR

	eGFR (mL/min/1.73 m^2^)					*P* value
	< 60	60–74	75–89	90–104	≥ 105	
N	185	408	632	717	266	
Baseline characteristics						
Male gender (n)	85 (45.9)	245 (60.0)	399 (63.1)	423 (59.0)	154 (57.9)	0.001
Age (yr)	68.8 ± 8.2	66.2 ± 9.4	63.8 ± 10.6	57.7 ± 10.2	41.4 ± 11.1	< 0.001
Body mass index (kg/m^2^)	24.1 ± 3.2	24.3 ± 3.3	24.3 ± 3.2	24.2 ± 3.3	22.9 ± 3.9	< 0.001
EuroSCORE (logistic)	9.6 ± 10.1	6.6 ± 6.9	6.0 ± 6.7	4.6 ± 5.3	5.6 ± 7.9	< 0.001
Hematocrit (%)	36.3 ± 5.9	38.3 ± 5.0	39.0 ± 4.9	39.1 ± 4.6	39.0 ± 5.1	< 0.001
Creatinine (mg/dL)	1.2 ± 0.1	1.0 ± 0.1	0.9 ± 0.1	0.8 ± 0.1	0.7 ± 0.1	< 0.001
Bilirubin, total (mg/dL)	0.7 ± 0.5	0.7 ± 0.6	0.7 ± 0.4	0.6 ± 0.4	0.7 ± 0.5	0.802
Albumin (g/dL)	3.6 ± 0.5	3.7 ± 0.4	3.7 ± 0.5	3.7 ± 0.5	3.8 ± 0.6	0.002
Uric acid (mg/dL)	7.0 ± 2.0	6.2 ± 1.7	5.6 ± 1.7	5.2 ± 1.5	5.0 ± 1.5	< 0.001
C-reactive protein (mg/dL)	0.8 ± 2.4	0.5 ± 1.3	0.6 ± 1.4	0.5 ± 1.3	1.1 ± 2.7	0.209
Left ventricle ejection fraction (%)	55.2 ± 11.4	57.0 ± 10.7	58.1 ± 10.5	59.3 ± 9.9	58.7 ± 10.1	< 0.001
Diabetes mellitus	62 (33.5)	111 (27.2)	138 (21.8)	150 (20.9)	22 (8.3)	< 0.001
Hypertension	119 (64.3)	243 (59.6)	331 (52.4)	306 (42.7)	58 (21.8)	< 0.001
Congestive heart failure	18 (9.7)	36 (8.8)	47 (7.4)	48 (6.7)	13 (4.9)	0.225
Cerebrovascular disease	29 (15.7)	47 (11.5)	47 (7.4)	52 (7.3)	13 (4.9)	< 0.001
Peripheral vascular disease	21 (11.4)	48 (11.8)	50 (7.9)	54 (7.5)	43 (16.2)	< 0.001
Liver disease	10 (5.4)	17 (4.2)	28 (4.4)	40 (5.6)	10(3.8)	0.683
Chronic obstructive pulmonary disease	8 (4.3)	21 (5.1)	28 (4.4)	24 (3.3)	9 (3.4)	0.604
Dyslipidemia	147 (79.5)	333 (81.6)	510 (80.7)	581 (81.0)	180 (67.7)	< 0.001
Smoker, current	19 (10.3)	60 (14.7)	86 (13.6)	149 (20.8)	67 (25.2)	< 0.001
ACEI or ARB	120 (64.9)	219 (53.7)	283 (44.8)	305 (42.5)	93 (35.0)	< 0.001
β-blocker	97 (52.4)	208 (51.0)	295 (46.7)	278 (38.8)	96 (36.1)	< 0.001
Calcium channel blocker	100 (54.1)	200 (49.0)	304 (48.1)	286 (39.9)	76 (28.6)	< 0.001
Diuretics	111 (60.0)	210 (51.5)	276 (43.7)	255 (35.6)	86 (32.3)	< 0.001
Insulin	25 (13.5)	33 (8.1)	47 (7.4)	50 (7.0)	12 (4.5)	0.009
Oral hypoglycemic agent	48 (25.9)	92 (22.5)	117 (18.5)	116 (16.2)	18 (6.8)	< 0.001
Aspirin	84 (45.4)	194 (47.5)	280 (44.3)	271 (37.8)	58 (21.8)	< 0.001
Clopidogrel	48 (25.9)	109 (26.7)	162 (25.6)	160 (22.3)	24 (9.0)	< 0.001
Statins	107 (57.8)	241 (59.1)	326 (51.6)	347 (48.4)	69 (25.9)	< 0.001
Intraoperative data						
Type of surgery						
Coronary artery bypass grafting	36 (19.5)	114 (27.9)	184 (29.1)	196 (27.3)	37 (13.9)	< 0.001
Valve	86 (46.5)	181 (44.4)	291 (46.0)	347 (48.4)	134 (50.4)	0.531
Aorta	12 (6.5)	23 (5.6)	28 (4.4)	37 (5.2)	32 (12.0)	< 0.001
Combined	51 (27.6)	90 (22.1)	129 (20.4)	137 (19.1)	63 (23.7)	0.102
Off-pump surgery	25 (13.5)	85 (20.8)	146 (23.1)	149 (20.8)	31 (11.7)	< 0.001
Operation time (min)	327.3 ± 110.9	311.5 ± 101.4	311.4 ± 100.2	300.9 ± 94.2	316.6 ± 116.7	0.065
Cardiopulmonary bypass time (min)	133.7 ± 84.9	114.8 ± 80.0	115.7 ± 84.8	114.7 ± 81.0	136.6 ± 81.0	0.575
Total crystalloid (L)	1.9 ± 0.8	2.1 ± 1.1	2.0 ± 1.0	1.9 ± 0.9	1.9 ± 1.0	0.058
Total colloid (L)	0.6 ± 0.3	0.6 ± 0.3	0.6 ± 0.3	0.6 ± 0.3	0.5 ± 0.3	0.093
Packed red blood cell (unit)	1.4 ± 2.3	1.2 ± 1.8	1.2 ± 2.1	0.7 ± 1.4	1.1 ± 2.4	< 0.001
Fresh frozen plasma (unit)	1.3 ± 2.1	1.1 ± 2.1	1.1 ± 2.2	0.8 ± 1.8	1.5 ± 3.2	0.528
Use of platelet concentrate	66 (35.7)	113 (27.7)	177 (28.0)	139 (19.4)	86 (32.3)	< 0.001
Use of cryoprecipitate	22 (11.9)	46 (11.3)	70 (11.1)	56 (7.8)	38 (14.3)	0.034

Data are expressed as number of patients (%) or mean ± standard deviation

For comparisons, one-way analysis of variance or Kruskal–Wallis test for continuous variables and the χ2 test for categorical variables were used, as appropriate

*eGFR* estimated glomerular filtration rate, *EuroSCORE* European System for Cardiac Operative Risk Evaluation, *ACEI* angiotensin-converting enzyme inhibitor, *ARB* angiotensin receptor blocker

The abilities of preoperative eGFR and preoperative sCr, both assessed as continuous variables, to predict composite 90-day postoperative major complications were compared. For this, c-statistics (equivalent to the area under the ROC curve [AUC]) for each final multivariable logistic regression model, each with eGFR or sCr separately, were calculated. To evaluate the discrimination ability of preoperative eGFR and sCr for predicting composite 90-day postoperative major complications, the adjusted AUCs (i.e., the c-statistics) with 95% CIs were compared using method of comparing areas based on correlated U statistics described by Delong et al. [23].

All statistical analyses were performed using SAS 9.4 (SAS Institute Inc., Cary, NC, USA) and IBM SPSS Statistics 21.0 (IBM Corp., Armonk, NY, USA) software. All reported *P* values were two-sided, with *P* <  0.05 considered statistically significant.

## Results

**Fig. 1:**
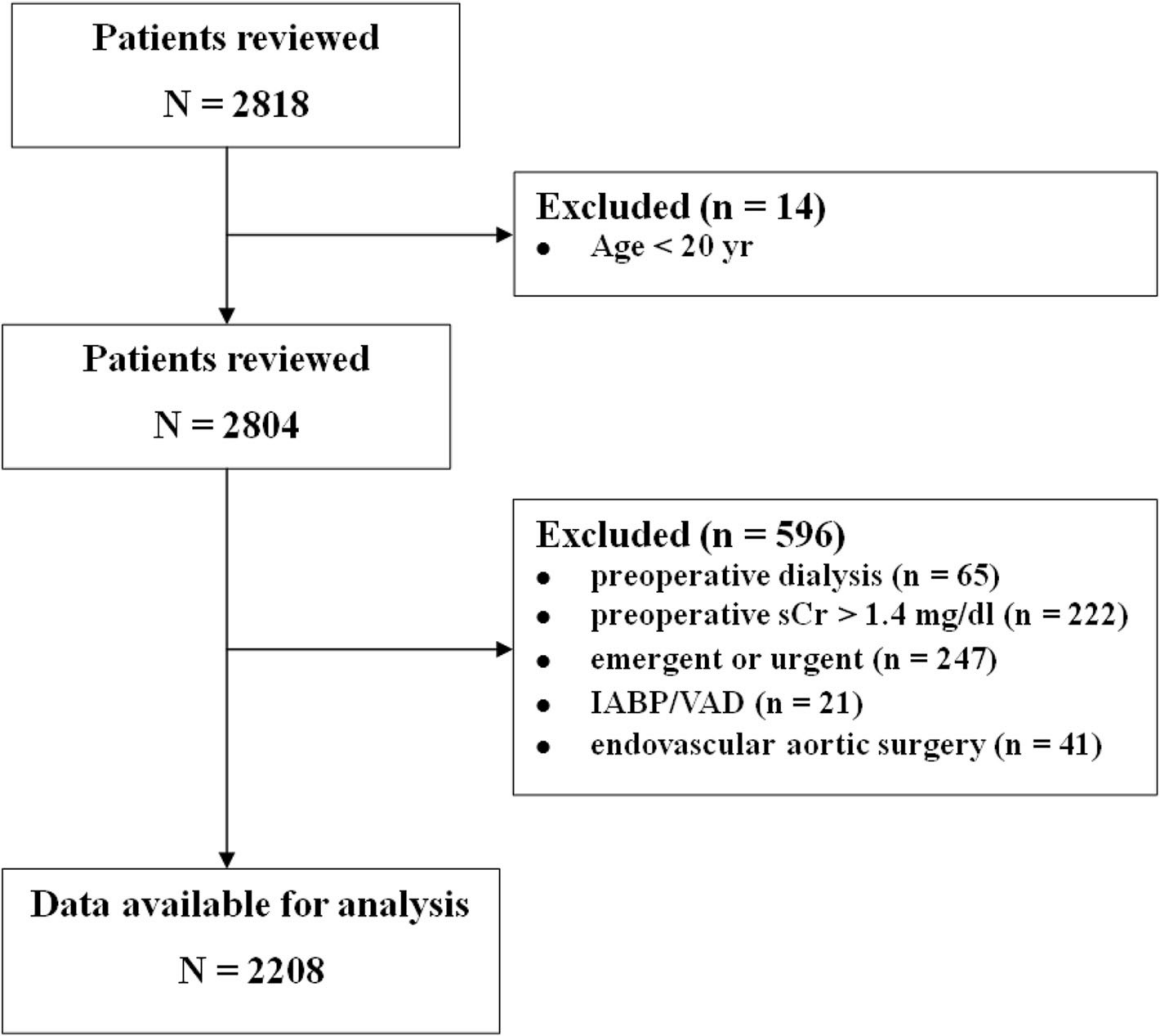
Flow diagram of the study, showing patients included and excluded. sCr = serum creatinine, IABP = intra-aortic balloon pump, VAD = ventricular assist device

During the study period, 2818 patients underwent cardiovascular surgery. After excluding 610 patients who met exclusion criteria, a total of 2208 patients were analyzed (Fig. 1). The baseline and intraoperative characteristics of the study population are shown in Table 1. Mean patient age was 60.0 ± 12.8 years, 59.1% were male, and mean preoperative sCr was 0.9 ± 0.2 mg/dL and mean preoperative eGFR was 86.3 ± 17.6 mL/min/1.73 m^2^ (Additional file 1: Table S1). Of the 2208 patients, 185 (8.4%) were found to have occult renal dysfunction (eGFR < 60 mL/min/1.73 m^2^), with the latter more likely in elderly than in younger patients and in women than in men. Despite strong negative correlations between sCr and eGFR in both males (Pearson correlation coefficient *R* = − 0.860; *P* <  0.001) and females (*R* = − 0.892; *P* <  0.001), the range of eGFR values among patients with low sCr was wide (Additional file 1: Figure S1).

**Fig. 2:**
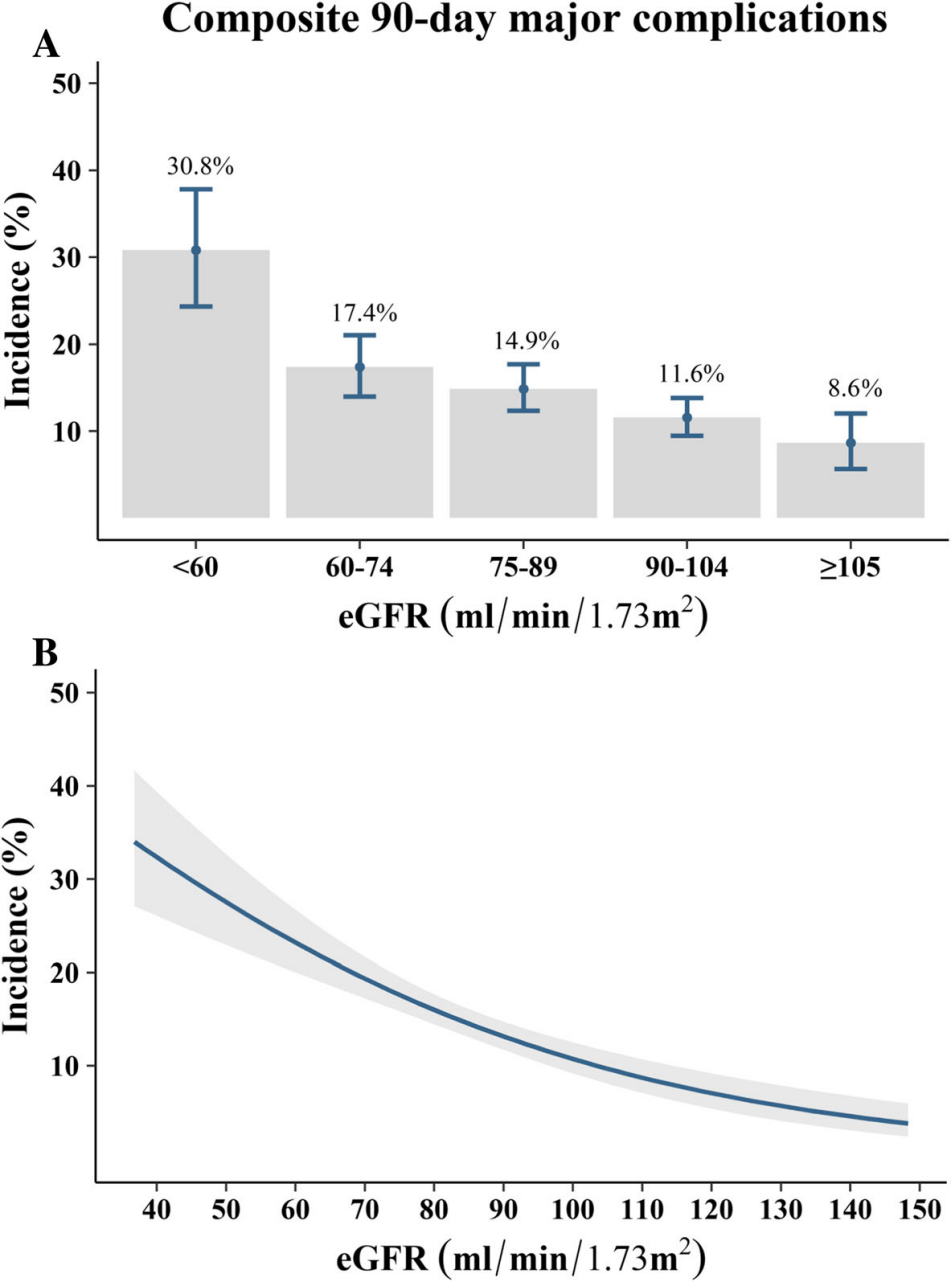
Relationship between preoperative eGFR and composite 90 day postoperative major complications as evaluated by (**a**) descriptive statistics and (**b**) logistic regression analysis. The 95% confidence intervals are denoted by error bars in **a** and bands around the regression line in **b**. eGFR = estimated glomerular filtration rate

**Fig. 3:**
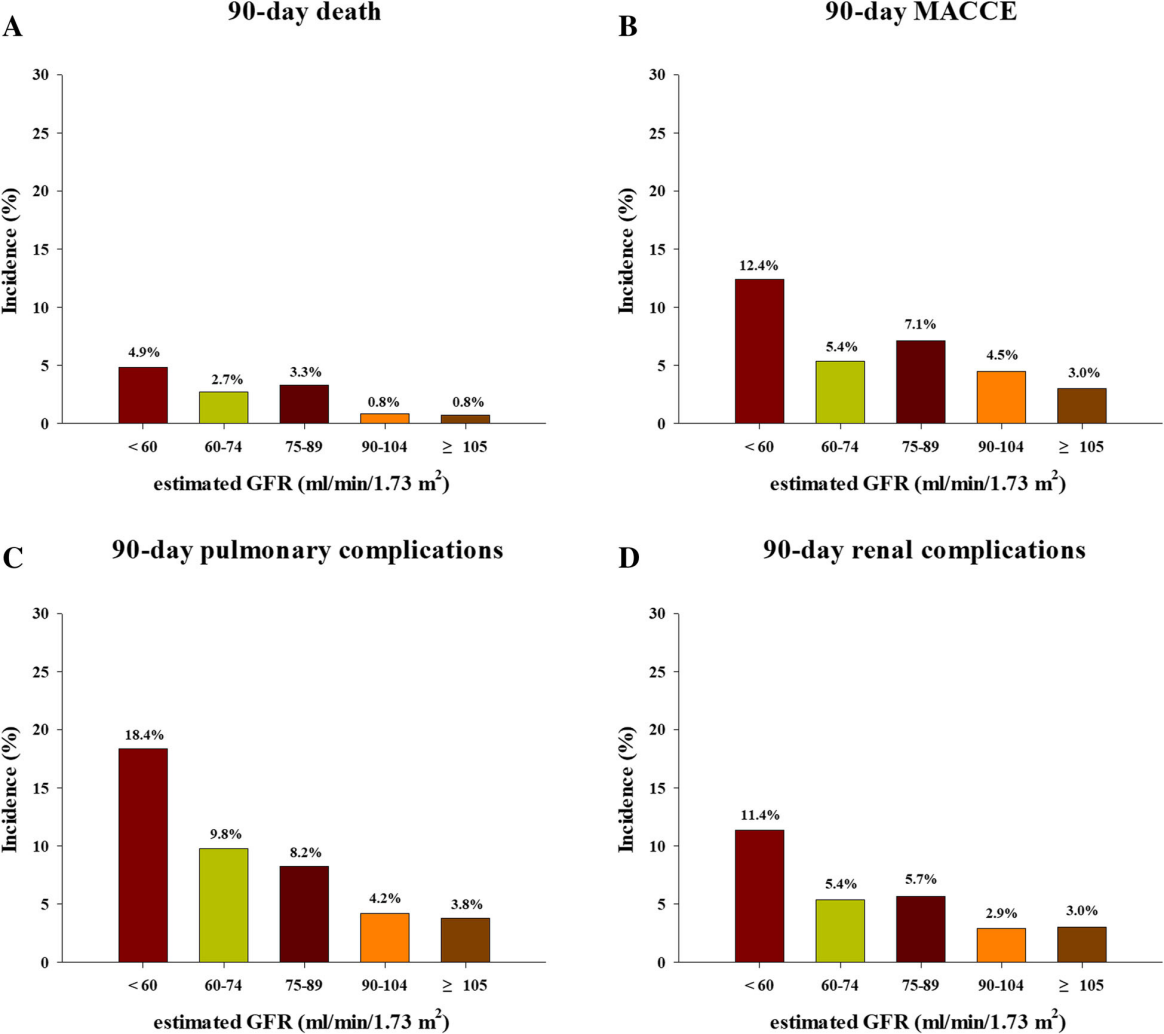
Effects of preoperative eGFR on the rates of 90-day (**a**) mortality, (**b**) MACCE, (**c**) pulmonary complications, and (**d**) renal complications after cardiovascular surgery. eGFR = estimated glomerular filtration rate; MACCE = major adverse cardiovascular and cerebrovascular event

Composite 30-day and 90-day postoperative major complications occurred in 296 (13.4%) and 328 patients (14.9%), respectively (Additional file 1: Table S2). Classification of the study population into five eGFR categories showed that the incidence of composite 90-day postoperative major complications increased from higher to lower eGFR categories (Fig. 2a). Similarly, when restricted cubic splines and logistic regression were used to analyze the relationship between eGFR as a continuous variable and composite 90-day postoperative major complications, an inverse relationship was observed (Fig. 2b). In addition, the 30-day and 90-day mortality, major adverse cardiovascular or cerebrovascular event, pulmonary, and renal complication rates increased from higher to lower eGFR categories, with all being particularly high in patients with eGFR < 60 mL/min/1.73 m^2^ (Fig. 3 and Additional file 1: Figure S2).

Unadjusted univariate analyses showed that factors significantly associated with composite 90-day postoperative major complication rates included patient age; body mass index; logistic EuroSCORE; sCr concentration; eGFR; hematocrit; concentrations of total bilirubin, serum albumin, uric acid, and C-reactive protein; left ventricular ejection fraction; congestive heart failure; peripheral vascular disease; liver disease; dyslipidemia; current smoker; and preoperative use of an angiotensin-converting enzyme inhibitor or angiotensin receptor blocker, and of diuretics and insulin. All these variables were incorporated into full multivariable logistic regression model (Table 2). Using the backward elimination method of multivariable logistic regression model, with the pre-specified significance level for removing and keeping factors in the model set to 0.05, the following parameters showed independent and significant associations with an increased risk of developing composite 90-day postoperative major complications (Table 2): peripheral vascular disease, current smoker, preoperative eGFR, hematocrit levels, serum total bilirubin levels, albumin levels, and preoperative use of diuretics.

**Table 2:** Univariate and multivariable predictors for composite 90-day major complications after cardiovascular surgery

Predictor	Univariate^a^		Multivariable^b^	
	OR (95% CI)	*P* value	OR (95% CI)	*P* value
eGFR^c^	1.259 (1.176–1.347)	< 0.001	1.232 (1.148–1.322)	< 0.001
Age	1.028 (1.017–1.038)	< 0.001		
Body mass index (kg/m^2^)	0.965 (0.931–1.000)	0.052		
EuroSCORE (logistic)	1.070 (1.054–1.086)	< 0.001		
Hematocrit (%)	0.908 (0.887–0.929)	< 0.001	0.963 (0.937–0.989)	0.005
Bilirubin, total (mg/dL)	1.514 (1.228–1.868)	< 0.001	1.368 (1.090–1.717)	0.007
Serum albumin (g/dL)	0.278 (0.219–0.352)	< 0.001	0.363 (0.276–0.478)	< 0.001
Uric acid (mg/dL)	1.101 (1.032–1.175)	0.003		
C-reactive protein (mg/dL)	1.249 (1.175–1.327)	< 0.001		
LVEF (%)	0.980 (0.970–0.990)	< 0.001		
Congestive heart failure	2.077 (1.427–3.023)	< 0.001		
Peripheral vascular disease	1.801 (1.295–2.538)	0.001	1.882 (1.295–2.736)	0.001
Liver disease	1.753 (1.094–2.809)	0.020		
Dyslipidemia	0.719 (0.547–0.944)	0.018		
Smoker, current	1.622 (1.149–2.290)	0.001	1.561 (1.126–2.165)	0.008
ACEI or ARB	1.285 (1.016–1.625)	0.036		
Insulin	1.989 (1.368–2.890)	< 0.001		
Use of diuretics	2.084 (1.643–2.643)	< 0.001	1.562 (1.198–2.037)	0.001

^a^ all variables were initially entered into a full multivariable regression analysis and were removed individually to assess each variable’s contribution to the model using backward elimination process with a *P* value cutoff of 0.05

^b^ final model, Hosmer-Lemeshow test; *P* = 0.738, C statistic = 0.724

^c^ for each 10 mL/min/1.73 m^2^ decrease

*OR* Odds Ratio, *CI* confidence interval, *eGFR* estimated glomerular filtration rate, *EuroSCORE* European System for Cardiac Operative Risk Evaluation, *LVEF* Left ventricle ejection fraction, *ACEI* angiotensin-converting enzyme inhibitor, *ARB* angiotensin receptor blocker

Multivariable logistic regression analyses showed that preoperatively decreased eGFR was independently associated with increased risk of composite 90-day postoperative major complications, with a 23% increased risk for each 10 mL/min/1.73 m^2^ reduction in eGFR (OR: 1.232; 95% CI: 1.148–1.322; *P* <  0.001). Compared with patients with preoperative eGFR ≥105 mL/min/1.73 m^2^, those in the lower eGFR categories, with eGFR < 60 mL/min/1.73 m^2^ (OR: 4.476; 95% CI: 2.542–7.883; *P* <  0.001), eGFR 60–74 mL/min/1.73 m^2^ (OR: 2.514; 95% CI: 1.482–4.264; *P* = 0.001), eGFR 75–89 mL/min/1.73 m^2^ (OR: 2.225; 95% CI: 1.336–3.706; *P* = 0.002), and eGFR 90–104 mL/min/1.73 m^2^ (OR: 1.687; 95% CI: 1.011–2.815; *P* = 0.045), were at significantly increased risk of composite 90-day postoperative major complications. In additional analyses with secondary outcome variables, similar results were obtained (Table 3).

**Table 3:** The odds ratios of preoperative eGFR for the various complications

	Unadjusted		Multivariable Adjusted	
	Odds Ratio (95% CI)^i^	*P* value	Odds Ratio (95% CI)^i^	*P* value
90-day death^a^	1.355 (1.153–1.592)	< 0.001	1.253 (1.072–1.465)	0.005
90-day MACCE^b^	1.224 (1.106–1.353)	< 0.001	1.182 (1.068–1.309)	0.001
90-day pulmonary complications^c^	1.360 (1.240–1.490)	< 0.001	1.238 (1.119–1.369)	< 0.001
90-day renal complications^d^	1.281 (1.147–1.430)	< 0.001	1.194 (1.064–1.339)	0.002
30-day composite complications^e^	1.355 (1.153–1.592)	< 0.001	1.253 (1.072–1.465)	0.005
30-day MACCE^f^	1.192 (1.072–1.326)	0.001	1.157 (1.040–1.288)	0.008
30-day pulmonary complications^g^	1.349 (1.229–1.481)	< 0.001	1.267 (1.151–1.394)	< 0.001
30-day renal complications^h^	1.288 (1.151–1.441)	< 0.001	1.208 (1.078–1.353)	0.001

^a^ adjusted by history of dyslipidemia, preoperative serum albumin levels, preoperative EuroSCORE (logistic), and preoperative use of diuretics

^b^ adjusted by history of liver disease, current smoking, preoperative EuroSCORE (logistic), preoperative serum total bilirubin and albumin levels, preoperative ejection fraction, preoperative use of statin, and preoperative use of ACEI or ARB

^c^ adjusted by history of peripheral vascular disease and liver disease, current smoking, preoperative EuroSCORE (logistic), preoperative hematocrit, preoperative serum albumin and uric acid levels, and preoperative use of diuretics

^d^ adjusted by history of hypertension, preoperative EuroSCORE (logistic), preoperative hematocrit, preoperative serum total bilirubin and albumin levels, preoperative ejection fraction, preoperative use of statin, and preoperative use of diuretics

^e^ adjusted by history of peripheral vascular disease and liver disease, current smoking, preoperative EuroSCORE (logistic), preoperative hematocrit, preoperative serum total bilirubin and albumin levels, and preoperative use of diuretics

^f^ adjusted by history of liver disease, current smoking, preoperative EuroSCORE (logistic), preoperative serum total bilirubin and albumin levels, preoperative use of statin, and preoperative use of ACEI or ARB

^g^ adjusted by history of peripheral vascular disease and liver disease, current smoking, preoperative EuroSCORE (logistic), preoperative hematocrit, preoperative serum albumin levels, and preoperative use of diuretics

^h^ adjusted by preoperative EuroSCORE (logistic), preoperative hematocrit, preoperative serum total bilirubin and albumin levels, preoperative ejection fraction, and preoperative use of diuretics

^i^ for each 10 U increase in the scale

*eGFR* estimated glomerular filtration rate, *CI* confidence interval, *MACCE* major adverse cardiovascular and cerebrovascular event, *EuroSCORE* European System for Cardiac Operative Risk Evaluation, *ACEI* angiotensin-converting enzyme inhibitor, *ARB* angiotensin receptor blocker

Although sCr was also effective for the prediction of composite 90-day postoperative major complications (OR: 3.871; 95% CI: 2.147–6.979; *P* <  0.001), eGFR was more accurate, as shown by their adjusted AUCs of 0.724 (95% CI: 0.694–0.754) for eGFR and 0.712 (95% CI: 0.680–0.744) for sCr (*P* = 0.008).

## Discussion

This retrospective observational study of 2208 patients who underwent cardiovascular surgery found that, even if sCr concentration is normal, GFR estimated by the CKD-EPI equation is a significant predictor of composite 90-day postoperative major complications and that this relationship is maintained despite adjustment for potential confounding variables. Furthermore, eGFR as a measure of renal function showed greater accuracy than sCr concentrations in multivariable risk models predicting composite 90-day postoperative major complications.

Regardless of how it is measured, renal dysfunction is an important predictor of postoperative adverse outcomes [3, 5, 7]. Previous studies, however, have found that assessing renal function only by dichotomized sCr levels may underestimate renal dysfunction, as sCr can be normal in patients with impaired renal function [10, 11]. Indeed, studies have shown that patients with normal sCr levels may have renal dysfunction that may affect postoperative outcome [1, 2, 12, 13]. For example, a study of 4603 patients with normal sCr undergoing cardiac surgery found that 565 (12.3%) had creatinine clearance, as estimated by the Cockroft-Gault equation, of < 60 mL/min/1.73 m^2^, which was related to increased risks of renal failure requiring dialysis, mortality, and major morbidity [1]. Another study showed that 13% of patients with normal sCr had estimated creatinine clearance < 60 mL/min/1.73 m^2^, which was associated with a nearly 3-fold increase in the risk of renal replacement therapy after cardiac surgery [12]. A third report found that 706 (8.2%) of 8562 patients with normal sCr had estimated creatinine clearance < 60 mL/min/1.73 m^2^, which was associated with higher risks for mortality and prolonged hospital stay after coronary artery bypass surgery [13]. More recently, a study found that approximately 40% of 9159 patients with normal sCr levels undergoing coronary artery bypass surgery had estimated creatinine clearance < 60 mL/min/1.73 m^2^ and that this factor was an independent predictor of mortality, renal dysfunction, dialysis, stroke, arrhythmia, and prolonged hospital stay [2]. Consistent with these previous reports, our study found that 8.4% of patients with normal sCr levels had eGFR < 60 mL/min/1.73 m^2^ and that this factor was significantly associated with an increased risk of composite 90-day postoperative major complications.

Thus, the results of our analyses confirm and extend the observations of previous studies that using eGFR with sCr rather than sCr alone may be more beneficial in clinical practice. Our study suggests that clinicians should use eGFR for evaluating preoperative renal function in patients undergoing cardiovascular surgery instead of relying on sCr value alone and that patients with occult renal dysfunction (eGFR <  60 mL/min/1.73 m^2^ despite having normal sCr values) should be considered as having clinically significant renal dysfunction linked to poor postoperative outcomes. In addition, the incorporation of this strategy in the preoperative assessment would facilitate the identification of high-risk patients who could remain unrecognized by clinicians when relying on sCr abnormalities alone to identify renal dysfunction and would provide better risk stratification that can help optimize monitoring and care strategies during the perioperative period.

In contrast to previous studies, which used the Cockroft-Gault equation to calculate eGFR, our study used the CKD-EPI equation. Although the Cockroft-Gault equation has been still used to determine the level of renal function, the CKD-EPI equation has been reported to be superior to the Cockroft-Gault equation in terms of eGFR accuracy and classification in several populations, particularly those with preserved renal function [24]. Moreover, the KDIGO clinical practice guidelines recommend using the sCr-based CKD-EPI equation for detecting and determining the severity of renal dysfunction, and for assessing the effects of treatment [17]. Because most of our patients had high eGFR levels, the use of the CKD-EPI equation to estimate GFR may strengthen the reliability of our findings. Our study also showed an inverse relationship between eGFR and the incidence of composite 90-day postoperative major complications, even when eGFR was greater than 60 mL/min/1.73 m^2^. Additionally, in agreement with previous studies [1, 12–14], our study showed that eGFR had a greater sensitivity than sCr in predicting postoperative outcomes. Taken together, these findings suggest that GFR estimated by the CKD-EPI equation may be useful for identifying patients with renal dysfunction undergoing cardiovascular surgery among patients thought to have preoperative normal renal function based on low sCr alone.

There are several limitations to be considered in the interpretation of our results. First, although our analyses included many variables, the retrospective and observational nature of this study may have masked hidden or unknown factors that may have influenced our results. Second, although the CKD-EPI equation has the highest accuracy in estimating GFR, GFR was not directly measured using any reference method or markers of kidney damage, including albuminuria. Thus, we cannot definitively conclude that actual preoperative renal function is directly associated with postoperative outcomes. Third, even though strenuous efforts have been made to achieve complete follow-up for all patients and to make our database as complete as possible, it is almost impossible to achieve 100% follow-up of all eligible subjects in a large-cohort study. Thus, in this study, we cannot completely rule out the possibility that complications suffered after discharge that were managed in primary care or local hospitals were missed, which may weaken the validity of our study. Accordingly, our results should be interpreted with caution. Finally, this was a single-center study and almost exclusively included Korean patients. Indeed, the CKD-EPI equation used to estimate GFR in this study was developed in a population comprising 99% Westerners (62% Caucasians, 32% African–Americans, and 5% Hispanics) and only 1% Asians [15], and its validity in Koreans and other Asian populations needs to be established. However, recent studies conducted in Asia on validating or establishing GFR estimating equations showed that the original CKD-EPI equation could be valid for evaluating the Korean and multiethnic Asian populations [25–27]. However, because we cannot completely exclude the possibility that the effect of race and ethnicity on estimating GFR could have influenced the results of this study, caution should be exercised in generalizing these results to centers with different patient populations.

## Conclusion

In conclusion, eGFR calculated using the CKD-EPI equation was significantly associated with composite 90-day postoperative major complications and has a significant advantage over sCr as a predictor of major complications after cardiovascular surgery in patients with normal sCr. These findings suggest that accurately assessing preoperative renal function by calculating eGFR in addition to measuring sCr may better identify patients at high risk for major complications after cardiovascular surgery and may be better for risk stratification.

## Additional file

Additional file 1: **Table S1.** Baseline and perioperative characteristics of the patient population. **Table S2.** Postoperative 30-day and 90-day complications. **Figure S1.** Correlation between preoperative serum creatinine concentration and eGFR calculated by the Chronic Kidney Disease Epidemiology Collaboration equation (*R* = –0.860, *P* < 0.001 in males; *R*= –0.892, *P* < 0.001 in females). eGFR = estimated glomerular filtration rate. **Figure S2.** Effects of preoperative eGFR on rates of 90 day (A) mortality, (B) MACCE, (C) pulmonary complications, and (D) renal complications (D) after cardiovascular surgery. eGFR = estimated glomerular filtration rate; MACCE = major adverse cardiovascular and cerebrovascular event. (PDF 464 kb)

## Data Availability

All data are available from the corresponding author upon reasonable request.

## Abbreviations

AUC: Areas under the ROC curve
CI: Confidence interval
CKD-EPI: Chronic Kidney Disease Epidemiology Collaboration
eGFR: Estimated glomerular filtration rate
KDIGO: Kidney Disease Improving Global Outcomes
OR: Odds ratio
ROC: Receiver operating characteristic
sCr: Serum creatinine
