# Supplementary material for: The relationship of preoperative estimated glomerular filtration rate and outcomes after cardiovascular surgery in patients with normal serum creatinine: a retrospective cohort study
Source: BMC Anesthesiol. 2019 May 29;19:88. doi: 10.1186/s12871-019-0763-1 (PMC6540432; doi:10.1186/s12871-019-0763-1)
Supplement: Supplementary file 1 — Table S1. Baseline and perioperative characteristics of the patient population. Table S2. Postoperative 30-day and 90-day complications. Figure S1. Correlation between preoperative serum creatinine concentration and eGFR calculated by the Chronic Kidney Disease Epidemiology Collaboration equation (R = –0.860, P < 0.001 in males; R= –0.892, P < 0.001 in females). eGFR = estimated glomerular filtration rate. Figure S2. Effects of preoperative eGFR on rates of 90 day (A) mortality, (B) MACCE, (C) pulmonary complications, and (D) renal complications (D) after cardiovascular surgery. eGFR = estimated glomerular filtration rate; MACCE = major adverse cardiovascular and cerebrovascular event. (PDF 464 kb) [file 12871_2019_763_MOESM1_ESM.pdf]

-Supplementary Materials-

The relationship of preoperative estimated glomerular filtration rate and outcomes after cardiovascular surgery in patients with normal serum creatinine: a retrospective cohort study

Myung-Soo Jang, Jae-Sik Nam, Jun-Young Jo, Chang-Hwa Kang, Seung Ah Ryu, Eun-Ho Lee, In-Cheol Choi

Table of Contents

Table S1. Baseline and perioperative characteristics of the patient population

Table S2. Postoperative 30-day and 90-day complications

Figure S1. Correlation between preoperative serum creatinine concentration and eGFR calculated by the Chronic Kidney Disease Epidemiology Collaboration equation (R = –0.860, P < 0.001 in males; R = –0.892, P < 0.001 in females). eGFR = estimated glomerular filtration rate.

Figure S2. Effects of preoperative eGFR on rates of 90 day (A) mortality, (B) MACCE, (C) pulmonary complications, and (D) renal complications (D) after cardiovascular surgery. eGFR = estimated glomerular filtration rate; MACCE = major adverse cardiovascular and cerebrovascular event.

Table S1. Baseline and perioperative characteristics of the patient population

Variables	Missing	Total	CPMC	No CPMC
N		2208	328	1880
Baseline characteristics				
Female (n, %)	0	902 (40.9)	148 (45.1)	754 (40.1)
Age (yr)	0	60.0 ± 12.8	63.4 ± 12.1	59.4 ± 12.8
Body mass index (kg/m^2)	1	24.1 ± 3.3	23.8 ± 3.7	24.2 ± 3.3
EuroSCORE (logistic)	0	5.9 ± 7.0	9.7 ± 10.5	5.3 ± 5.9
Hematocrit (%)	0	38.7 ± 5.0	36.6 ± 5.9	39.0 ± 4.7
Creatinine (mg/dL)	0	0.9 ± 0.2	0.9 ± 0.2	0.9 ± 0.2
eGFR (mL/min/1.73 m^2)	0	86.3 ± 17.6	80.3 ± 18.6	87.4 ± 17.2
Bilirubin, total (mg/dL)	1	0.7 ± 0.5	0.8 ± 0.7	0.7 ± 0.4
Albumin (g/dL)	1	3.7 ± 0.5	3.5 ± 0.6	3.8 ± 0.4
Uric acid (mg/dL)	2	5.6 ± 1.7	5.9 ± 2.1	5.6 ± 1.7
C-reactive protein (mg/dL)	77	0.6 ± 1.7	1.4 ± 2.9	0.5 ± 1.3
Left ventricle ejection fraction (%)	2	58.1 ± 10.4	56.0 ± 12.0	58.5 ± 10.1
Diabetes mellitus	0	483 (21.9)	80 (24.4)	403 (21.4)
Hypertension	0	1057 (47.9)	170 (51.8)	887 (47.2)
Congestive heart failure	0	162 (7.3)	41 (12.5)	121 (6.4)
Cerebrovascular disease	0	188 (8.5)	36 (11.0)	152 (8.1)
Peripheral vascular disease	0	216 (9.8)	49 (14.9)	167 (8.9)
Liver disease	0	105 (4.8)	24 (7.3)	81 (4.3)
COPD	0	90 (4.1)	15 (4.6)	75 (4.0)
Dyslipidemia	0	1751 (79.3)	244 (74.4)	1507 (80.2)
Smoker, current	0	381 (17.3)	64 (19.5)	317 (16.9)
ACEI or ARB	0	1020 (46.2)	169 (51.5)	851 (45.3)
β-blocker	0	974 (44.1)	152 (46.3)	822 (43.7)
Calcium channel blocker	0	966 (43.8)	153 (46.6)	813 (43.2)
Diuretics	0	938 (42.5)	190 (57.9)	748 (39.8)
Insulin	0	167 (7.6)	41 (12.5)	126 (6.7)
Oral hypoglycemic agent	0	391 (17.7)	65 (19.8)	326 (17.3)
Aspirin	0	887 (40.2)	127 (38.7)	760 (40.4)
Clopidogrel	0	503 (22.8)	74 (22.6)	429 (22.8)

Statins	0	1090 (49.4)	154 (47.0)	936 (49.8)
Intraoperative data				
Type of surgery				
Coronary artery bypass grafting	0	567 (25.7)	57 (17.4)	510 (27.1)
Valve	0	1039 (47.1)	137 (41.8)	902 (48.0)
Aorta	0	132 (6.0)	40 (12.2)	92 (4.9)
Combined	0	470 (21.3)	94 (28.7)	376 (20.0)
Off-pump surgery	0	436 (19.7)	39 (11.9)	397 (21.1)
Operation time (min)	0	310.0 ± 101.8	371.0 ± 131.4	299.3 ± 91.7
CPB time (min)	0	119.2 ± 82.6	156.0 ± 96.7	112.8 ± 78.1
Total crystalloid (L)	0	2.0 ± 1.0	2.2 ± 1.1	1.9 ± 0.9
Total colloid (L)	0	0.6 ± 0.3	0.6 ± 0.3	0.6 ± 0.3
Packed red blood cell (unit)	0	1.1 ± 1.9	2.3 ± 3.2	0.8 ± 1.5
Fresh frozen plasma (unit)	0	1.1 ± 2.2	2.5 ± 3.3	0.8 ± 1.9
Use of platelet concentrate	0	581 (26.3)	159 (48.5)	422 (22.4)
Use of cryoprecipitate	0	232 (10.5)	71 (21.6)	161 (8.6)
Extubation time (hr)	0	9 [6–14]	21 [11–85]	9 [6–13]
ICU stay (hr)	0	45 [28–65]	116 [52–260]	44 [26–49]
Hospital stay (d)	0	8 [7–12]	19 [11–39]	8 [7–11]

Data are expressed as number of patients (%), mean ± standard deviation, or median [first-third quartiles].

CPMC = composite 90-day postoperative major complications; eGFR = estimated glomerular filtration rate; EuroSCORE = European System for Cardiac Operative Risk Evaluation; COPD = chronic obstructive pulmonary disease; ACEI = angiotensin-converting enzyme inhibitor; ARB = angiotensin receptor blocker; CPB = cardiopulmonary bypass; ICU = intensive care unit.

Table S2. Postoperative 30-day and 90-day complications

Complications	Frequency, n (%)	
	**30-day**	**90-day**
MACCE	115 (5.2)	130 (5.9)
Major adverse cardiovascular event	70 (3.2)	81 (3.7)
Ischemic or hemorrhagic stroke	56 (2.5)	77 (3.5)
Pulmonary complication	160 (7.2)	166 (7.5)
Mechanical ventilation > 48 h	127 (5.8)	127 (5.8)
Pneumonia	69 (3.1)	75 (3.4)
Acute lung injury or acute respiratory distress syndrome	6 (0.3)	7 (0.3)
Renal complication	104 (4.7)	108 (4.9)
≥ KDIGO stage2	94 (4.3)	94 (4.3)
Renal replacement therapy	58 (2.6)	62 (2.8)
Wound complication	55 (2.5)	69 (3.1)
Gastrointestinal complication	7 (0.3)	7 (0.3)
Sepsis	10 (0.5)	17 (0.8)
Multi-organ failure	7 (0.3)	10 (0.5)
In-hospital death	41 (1.9)	41 (1.9)
Death	28 (1.3)	49 (2.2)
Composite major complications	296 (13.4)	328 (14.9)

Data are expressed as number of patients (%) or median [interquartile range].

MACCE = major adverse cardiovascular and cerebrovascular event; KDIGO = Kidney Disease Improving Global Outcomes classification

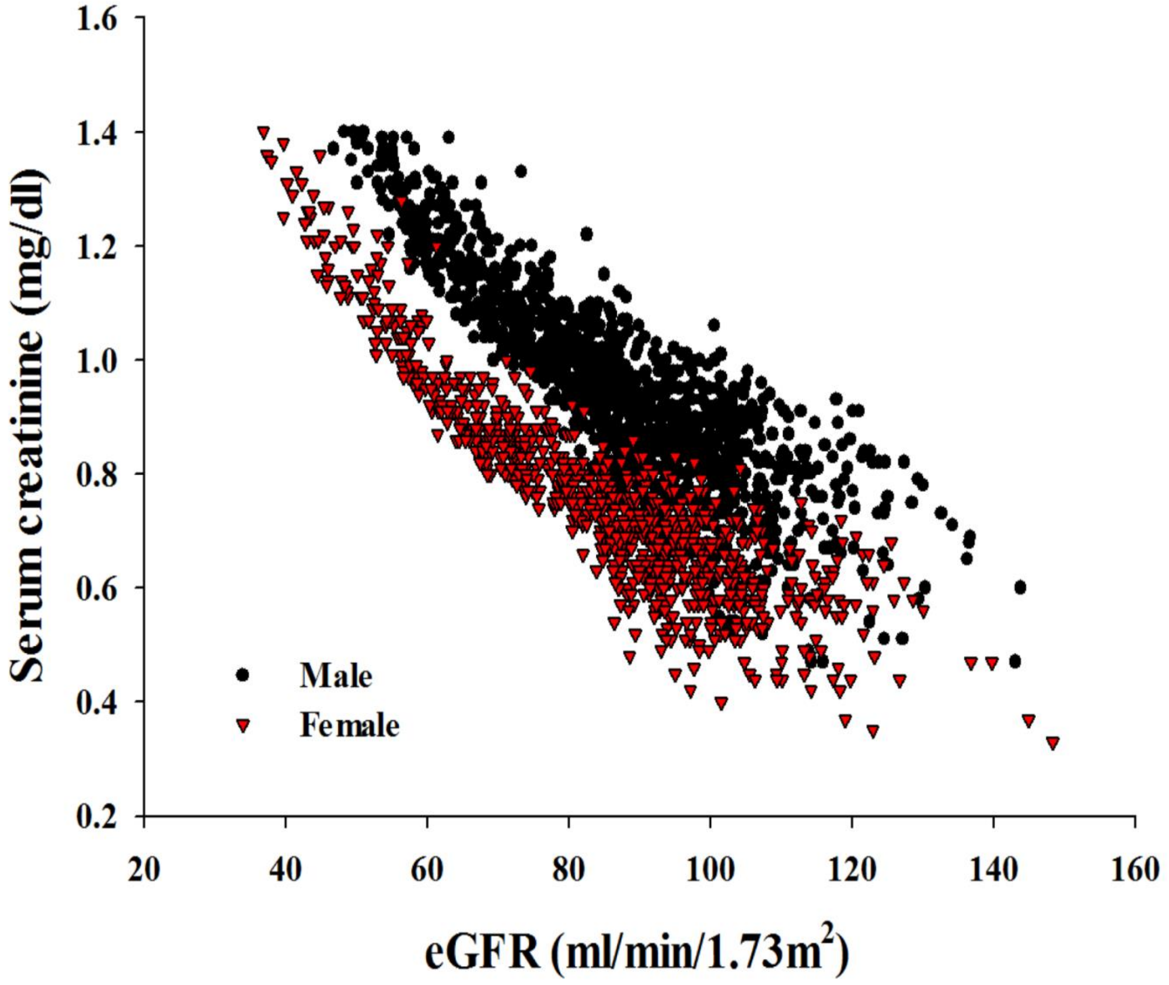

Figure S1. Correlation between preoperative serum creatinine concentration and eGFR calculated by the Chronic Kidney Disease Epidemiology Collaboration equation (R = –0.860, $P < 0.001$ in males; R = –0.892, $P < 0.001$ in females). eGFR = estimated glomerular filtration rate.

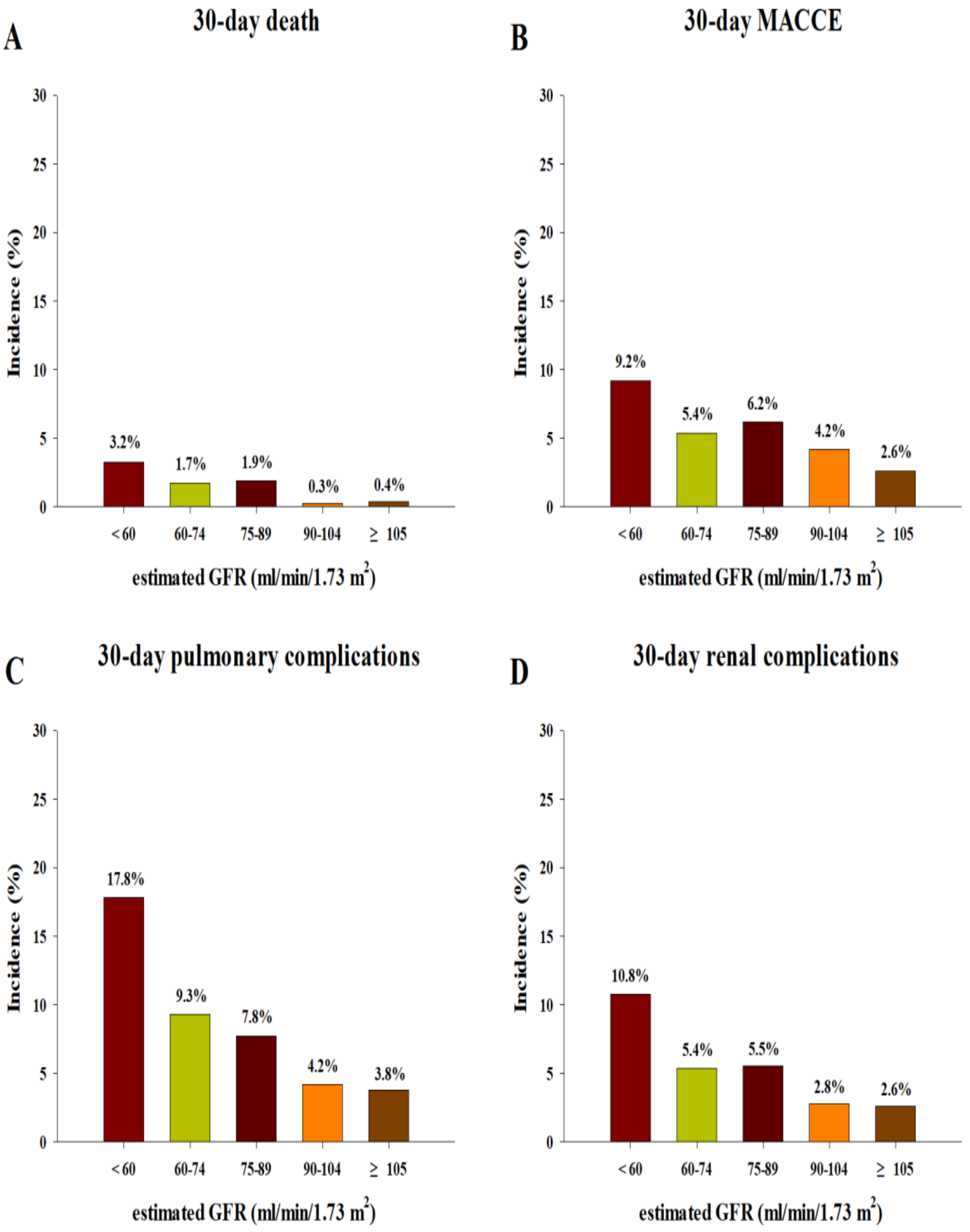

Figure S2. Effects of preoperative eGFR on rates of 30-day (A) mortality, (B) MACCE, (C) pulmonary complications, and (D) renal complications after cardiovascular surgery. eGFR = estimated glomerular filtration rate; MACCE = major adverse cardiovascular and cerebrovascular event.
